# Transcanal microscope-assisted endoscopic myringoplasty in children

**DOI:** 10.1186/s12887-015-0351-6

**Published:** 2015-04-01

**Authors:** Lela Migirov, Michael Wolf

**Affiliations:** Department of Otolaryngology Head and Neck Surgery, Sheba Medical Center, Affiliated to the Sackler School of Medicine, Tel Aviv University, Tel Aviv, Israel; Department of Otolaryngology and Head & Neck Surgery, Sheba Medical Center, Tel Hashomer, 5262l Israel

**Keywords:** Endoscopic, Children, Myringoplasty, Outcome, Surgery

## Abstract

**Background:**

Myringoplasty can be technically difficult in the pediatric patients due to the narrowness of the external auditory canal and the generally small size of the ear. Moreover, temporalis fascia grafts and myringoplasties for anterior perforations are more likely to fail in children. Surgical management of anterior perforations requires total exposure of the anterior angle however a microscope may fail to provide a view of the anterior edge in most of perforations. Recently, different endoscopes are used in the performance of ear surgery in general and myringoplasty in particular. Current study aimed to investigate the outcome of transcanal microscope-assisted endoscopic myringoplasty in homogenous group of children.

**Methods:**

The medical records of 22 children were retrospectively reviewed for age, perforation size and location, surgical and audiological findings, and outcome. All myringoplasties were performed by first author with a chondro-perichondrial graft that has been harvested from the tragus and placed medial to the tympanic membrane remnants, utilizing the underlay technique and 14-mm length, 3-mm diameter, 0° and 30° endoscopes. A microscope was occasionally used for removal of the sclerotic plaques and releasing adhesions surrounding the ossicles when bimanual manipulations were needed. Surgical success was defined as a tympanic membrane with no perforation, retraction, or graft lateralization for at least 18 months following surgery.

**Results:**

Thirteen large-, 8 medium- and 1 small-sized perforations (defined as 75, 50 or 25%, respectively, of the tympanic membrane area), of which 14 were anterior, 2 central and 6 posterior marginal, were repaired. The edges of the defect could not be visualized under a microscope due to bone overhanging or a curved or narrow EAC in 8 anterior perforations. Intact tympanic membranes and dry ears were achieved in all operated children. The audiometric air conduction level (average of 0.5-3 kHz) for the entire cohort ranged between 10–51.3 dB (mean 32.8) preoperatively and between 5–35 dB (mean 18.2) postoperatively.

**Conclusion:**

The transcanal microscope-assisted endoscopic myringoplasty had a 100% rate of surgical success in children. This technique can be especially appropriate for patients with narrow external canals, anterior defects and bone overhang making the perforation margins barely visible under a microscope.

## Background

Recently, different endoscopes are used in the performance of ear surgery in general and myringoplasty in particular, and the surgical success of endoscope-assisted myringoplasty ranges between 80 and 100% [1-8]. Most of the earlier studies investigating the surgical outcome of endoscopic or endoscope-assisted myringoplasties were heterogeneous by having included both children and adults, and by using different graft materials and various surgical techniques [1-6]. Myringoplasty can be technically difficult in the pediatric patients due to the narrowness of the external auditory canal and the generally small size of the ear [7,9,10]. Moreover, temporalis fascia grafts and myringoplasties for anterior perforations are more likely to fail in children [1-4,11,12]. Surgical management of anterior perforations requires total exposure of the anterior angle, but a microscope may fail to provide a view of the anterior edge in 73% of perforations that can, however, be entirely exposed with an endoscope [5]. As a result, drilling of the anterior part of an external auditory canal is usually unavoidable for the repair of anterior perforations when only a microscopic approach is employed [11].

The current study was designed to evaluate the surgical and audiological outcome of transcanal microscope-assisted endoscopic myringoplasty utilizing a chondro-perichondrial graft among a homogenous group of children.

## Methods

The medical records of children who underwent transcanal myringoplasty by the first author between 2009–2012 were reviewed for age, gender, perforation size (i.e., small, medium or large, defined as 25, 50 or 75%, respectively, of the area of the tympanic membrane), perforation location (anterior, central or posterior), surgical findings (i.e., myringosclerotic plaques and status of ossicular chain), audiological findings before and after surgery, i.e., air conduction, bone conduction and air–bone gap for 500, 1000, 2000 and 3000 Hz, and surgical outcome (i.e., closed tympanic membrane, residual perforation or re-perforation). The condition for myringoplastic surgery was dry ear and normal middle ear mucosa for at least 3 months. The Eustachian tube function in these children was assumed as suitable for myringoplastic surgery. It should be noted that the data on simple primary myringoplasty was analyzed. The outcome of surgeries that had been performed for ossicular chain problems is out of focus of the current paper.

**Figure 1:**
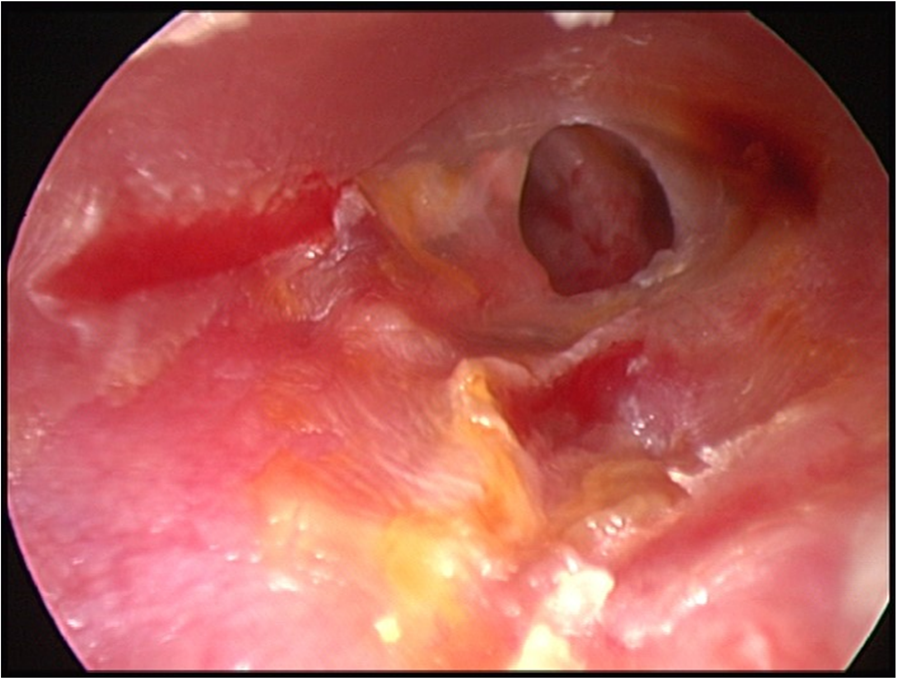
**Endoscopic view of a medium-sized perforation in the left tympanic membrane.**

**Figure 2:**
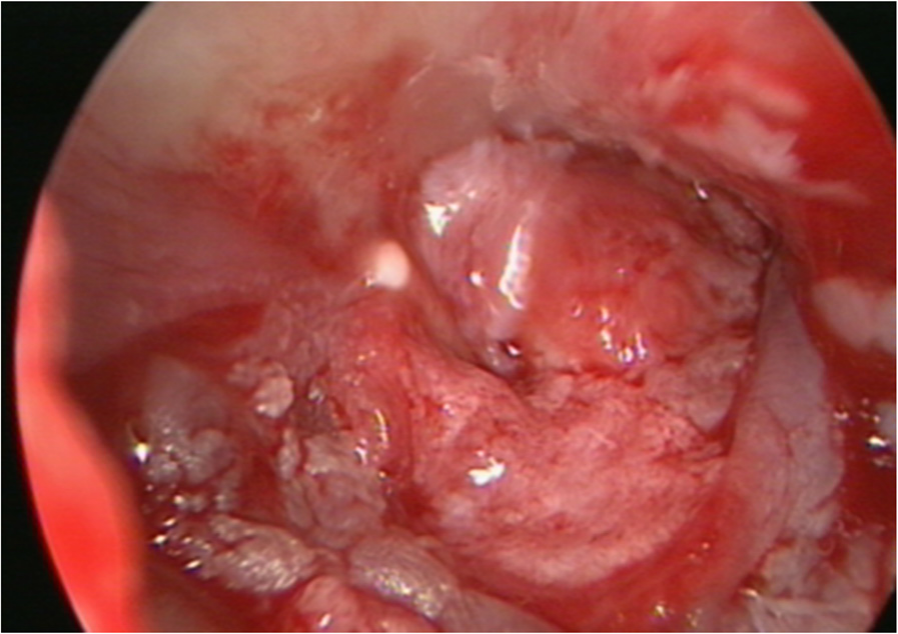
**Endoscopic view of the same ear as in Figure** **1** **at the end of the myringoplasty.**

All myringoplasties were performed under general anesthesia with a chondro-perichondrial graft that had been harvested from the tragus and placed medial to the tympanic membrane remnants, utilizing the underlay technique and 14-mm length, 3-mm diameter, 0° and 30° endoscopes (Figures 1 and 2). The external ear canal was injected with lidocaine 1% with 0.5:100.000 epinephrine. Tympanomeatal flap was elevated using the 0° endoscope in all the cases, and the 30° endoscope was utilized for better visualization of 4 anterior perforations. The margins of perforations were freshened using the 0° or 30°endoscopes. Microscope was used for removal of the sclerotic plaques and releasing adhesions surrounding the ossicles in 4 cases when bimanual manipulations were needed. The tympano-meatal flap was repositioned, and the external auditory canal was filled with Gelfoam® soaked in ear drops containing antibiotics.

Surgical success was defined as a tympanic membrane with no perforation, retraction, or graft lateralization for at least 18 months following surgery. Post-operative audiogram was performed at 6–8 weeks and 1 year following surgery. The study was approved by the Institutional Review Board of the Sheba Medical Center.

## Results

The study group included 22 children (11 girls, 11 boys; age range 5 to 16 years, mean 10.7 years) with 13 large-, 8 medium- and 1 small-sized perforation of which 14 were anterior, 2 central and 6 posterior marginal defects. The edges of the defect could not be visualized under a microscope due to bone overhanging or a curved or narrow EAC in 8 anterior perforations. The endoscopes in patients with central and posterior marginal perforations were used mainly for training. Myringosclerotic plaques in the remnant tympanic membrane were observed and removed intra-operatively in 16 (72.7%) children. Three children had non-suppurative perforation with normal middle ear mucosa also in their contralateral ears. The other patients had closed tympanic membrane with well aerated middle ear cavity.

Intact tympanic membranes and dry ears were achieved in all operated children. There were no incidents of iatrogenic injuries to the facial nerve, chorda tympani or to the ossicles in this series.

The audiometric air conduction level (average of 0.5-3 kHz) for the entire cohort ranged between 10–51.3 dB (mean 32.8) preoperatively and between 5–35.8 dB (mean 18.2) postoperatively (Table 1). None of the study group patients demonstrated postoperative worsening of the bone conduction threshold.

**Table 1:** **Pre and postoperative hearing results for 22 operated children**

	**Preoperative results**			**12 months after surgery**		
	AC	BC	ABG	AC	BC	ABG
Range	10–51.3	5–38.1	5–20.4	5–35.8	5–35	0–15.2
Mean	32.8	21.7	11.3	18.2	18.0	1.5

AC-air conduction, BC-bone conduction, ABG-air-bone gap.

## Discussion

Transmeatal microscope-assisted endoscopic myringoplasty with an underlay chondro-perichondrial graft obtained 100% surgical success rate and good functional results in pediatric patients. Endoscopes enabled successful myringoplasty in children in whom the perforated edges of the tympanic membrane were invisible under a microscope. The microscope provided valuable assistance in terms of surgical accuracy when bimanual manipulations were required.

Our results can be compared with two other homogeneous studies on endoscopic myringoplasty [7,8]. Mohindra and Panda reported 100% surgical success rate of endoscopic myringoplasty with temporalis fascia grafting in 14 children aged 5–15 years [7]. Dündar et al. found 87.5% closure of perforation with endoscopic myringoplasty using a boomerang-shaped chondro-perichondrial graft in children aged 7–16 years [8].

We found that endoscope was very effective in ensuring satisfactory approximation of graft material to the perforation margins in large and subtotal perforations as well.

We believe that the rate of graft take in our study was higher than those reported in other studies on myringoplasty in children [7,11-15] due to use of both an endoscope and a microscope as well as the choice of and chondro-perichondrial graft material in our pediatric patients. The personal experience of the first author supports the hypothesis that removal of the sclerotic plaques from the remnant tympanic membrane may contribute to an excellent surgical outcome [16].

The relatively small number of participants might pose a limitation to the current study. The endoscopes are routinely used in oto-surgeries in our department since 2008, and we do not support the randomization of surgical approaches (endoscopic, endoscope-assisted microscopic, microscopic or microscope-assisted endoscopic) in current management of different ear pathologies. We considered the randomization of surgical approach for myringoplasty in children as unethical, thus there is no real control group for the presented series.

## Conclusions

The transcanal microscope-assisted endoscopic myringoplasty had a 100% rate of surgical success for closure of tympanic membrane defects in children. This technique is especially helpful in patients with narrow external canals, anterior defects and bone overhang, when perforation’s margins are barely, if at all, visible under a microscope. The choice of chondro-perichondrial graft material and the meticulous removal of myringosclerotic plaques can enhance the surgical outcome of pediatric myringoplasty performed by an experienced otologist.
